# The revised WHO dengue case classification: does the system need to be modified?

**DOI:** 10.1179/2046904712Z.00000000052

**Published:** 2012-05

**Authors:** Sri Rezeki S Hadinegoro

**Affiliations:** Department of Child Health, Faculty of Medicine, University of Indonesia, Dr Cipto Mangunkusumo Hospital, Jakarta, Indonesia

**Keywords:** Dengue, Classification, Diagnosis, World Health Organization

## Abstract

There has been considerable debate regarding the value of both the 1997 and 2009 World Health Organization (WHO) dengue case classification criteria for its diagnosis and management. Differentiation between classic dengue fever (DF) and dengue haemorrhagic fever (DHF) or severe dengue is a key aspect of dengue case classification. The geographic expansion of dengue and its increased incidence in older age groups have contributed to the limited applicability of the 1997 case definitions. Clinical experience of dengue suggests that the illness presents as a spectrum of disease instead of distinct phases. However, despite the rigid grouping of dengue into DF, DHF and dengue shock syndrome (DSS), overlap between the different manifestations has often been observed, which has affected clinical management and triage of patients. The findings of the DENCO study evaluating the 1997 case definitions formed the basis of the revised 2009 WHO case definitions, which classified the illness into dengue with and without warning signs and severe dengue. Although the revised scheme is more sensitive to the diagnosis of severe dengue, and beneficial to triage and case management, there remain issues with its applicability. It is considered by many to be too broad, requiring more specific definition of warning signs. Quantitative research into the predictive value of these warning signs on patient outcomes and the cost-effectiveness of the new classification system is required to ascertain whether the new classification system requires further modification, or whether elements of both classification systems can be combined.

## Introduction

Approximately 1 million cases of dengue, a major cause of morbidity in tropical and sub-tropical regions, are reported annually to the World Health Organization (WHO).^1^ The 2009 revised WHO dengue case classification for the diagnosis and management of the illness follows previous guidelines published by WHO between 1974 and 1997.1,^2^ This article investigates the clinical application of the 1997 and 2009 criteria to the reporting and management of dengue and the difficulties of using the classification schemes. The article also explores whether the changes to the 1997 guidelines have been beneficial and discusses whether the revised 2009 guidelines may benefit from further modification.

## Clinical Presentation of Dengue

Expert consensus groups have suggested that dengue is a single entity with different clinical presentations and infected patients present with a range of clinical symptoms that vary according to severity and age.^3^ Infection by any of the four dengue serotypes may be asymptomatic or lead to classic dengue fever (DF) or more severe forms of the disease, haemorrhagic fever (DHF) and dengue shock syndrome (DSS).^4^ Confirmation of dengue infection may be possible during the acute phase by testing the serum for presence of the non-structural protein (NS1) antigen.^5^ Following an incubation period, the illness begins abruptly, going through three phases: febrile, critical and recovery.^6^ DF is observed more frequently in adults and adolescents, and can present with either mild fever only or a more incapacitating disease. This latter presentation is characterised by the sudden onset of high fever, severe headache, retro-orbital pain, myalgia, arthralgia and rash,3,^7^ symptoms occurring predominantly in the early febrile stage.^8^ In the critical phase, the skin is flushed with the appearance of a petechial rash. This usually occurs around the time of defervescence, typically on days 3–7, and is associated with capillary leakage and haemorrhage.^4^

DHF or severe dengue usually affects children younger than 15 years, although it can occur in adults.^7^ DHF is characterised by a transient increase in vascular permeability resulting in plasma leakage, with high fever, bleeding, thrombocytopenia and haemoconcentration, which can lead to shock (termed dengue shock syndrome (DSS).^8^ However, it can be difficult to differentiate DHF from DF and other viral diseases, e.g. typhoid fever, particularly during the acute phase of the illness.^7^

**Figure 1:**
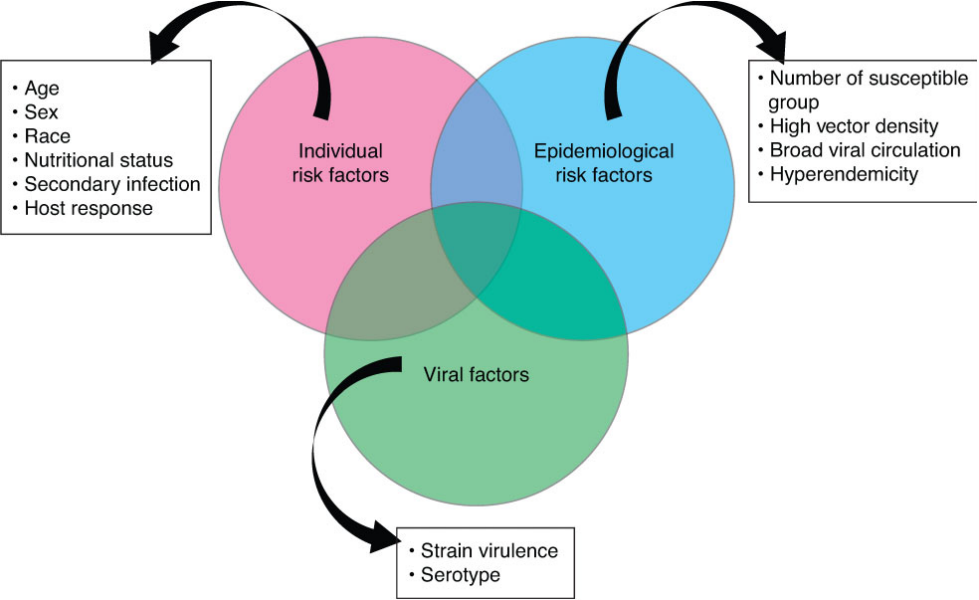
Risk factors for dengue haemorrhagic fever^8^

Secondary dengue infection is considered to be the principal risk factor for DHF, but the interaction of virus, host and epidemiological risk factors are determinants of the occurrence of DHF epidemics (Fig. 1).^8^

## WHO Dengue Case Classification

Central to dengue case classification is the differentiation between classic DF and DHF or severe dengue. For greater clarity on these distinctions, a WHO committee developed case classification guidelines in 1974, based on studies of disease patterns in children in Thailand in the 1960s, which were subsequently modified and published a number of times.^9^ However, it became apparent that this classification system is not universally applicable for appropriate clinical management, and in 2006 the WHO Dengue Scientific Working Group recommended additional research into dengue diagnostics and triaging of patients for optimised clinical management.^10^

Further studies on the use of clinical guidelines for dengue diagnosis, including the Dengue Control (DENCO) study, led to the re-classification of dengue into non-severe and severe cases.^10^ This was subsequently revised into dengue with and without warning signs and severe dengue, and was published in 2009.^6^

## The 1997 Dengue Case Classification

**Figure 2:**
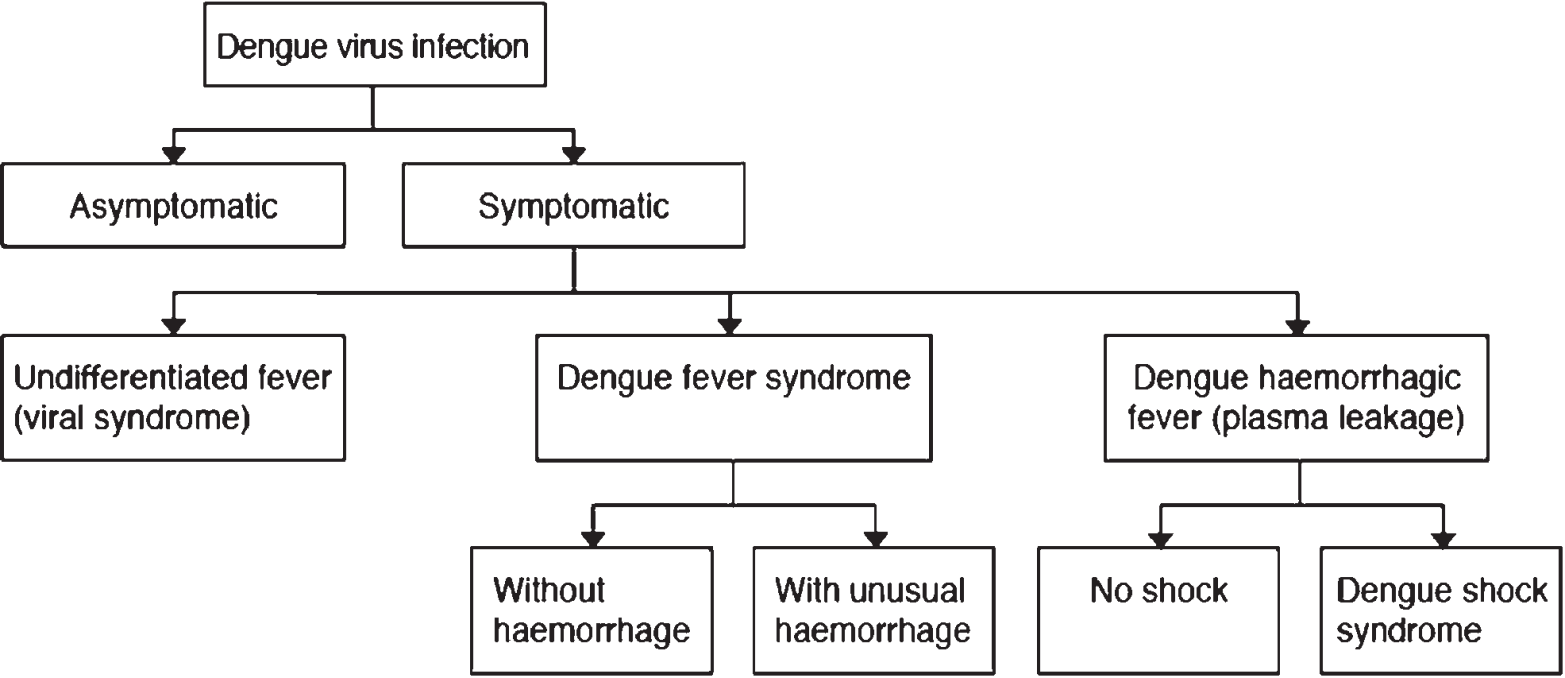
The 1997 WHO classification of dengue virus infection.^11^ Reprinted from Lancet, Deen JL, Harris E, Wills B, Balmaseda A, Hammond SN, Rocha C, *et al*, The WHO dengue classification and case definitions: time for a reassessment, 170–3, Copyright 2012, with permission from Elsevier

The 1997 guidelines (Fig. 2) classified dengue into DF, DHF (Grades 1 and 2) and DSS (DHF Grades 3 and 4).9,^11^ The case diagnosis for DF emphasised the need for laboratory confirmation and the suggested DF classifications are shown in Box 1, together with those for DHF and DSS.

Experience with this classification system has exposed a number of limitations. It is based on clinical data in Thai children, which may not be universally representative of dengue, following its expansion to additional tropical regions and older age groups.^12^ A range of clinical tests requiring repetition is also needed, which can be difficult for countries with limited resources to perform regularly.^13^ Integral to the 1997 case definitions is the tourniquet test, a measure of capillary fragility and thrombocytopenia, for the diagnosis of DHF. However, the test does not differentiate effectively between DF and DHF11,13,^14^ and dengue and other febrile illnesses.^11^

Studies have demonstrated overlap between case definitions of DF, DHF and DSS, supporting the concept of dengue as a continuous spectrum of disease rather than distinct entities.11,^14^ Another study found that strict application of the criteria did not detect severe dengue manifestations in many patients, particularly adults.^15^ Indeed, the term DHF is considered to place undue emphasis on haemorrhage when plasma leakage leading to shock is a more significant warning sign.^11^ Manifestations of severe dengue include organ failure, but this was not included in the 1997 case definitions.^2^ New classifications were developed to account for observed disease patterns.2,11,^16^

## The DENCO Study: Evaluating the 1997 Classification

The international DENCO study was designed to evaluate the perceived limitations of the 1997 criteria in all age groups in South-east Asia and Latin America to develop an evidence-based classification that would better reflect clinical severity.^17,18^

Over 1700 confirmed cases of dengue were categorised into one of three intervention groups according to disease severity. Potential warning signs were identified by comparing data of patients who did and did not progress to severe disease. The study found that 22% of patients with shock did not fulfil all the criteria for DHF.^17^ These results formed the basis of the revised 2009 WHO classification system.

## The 2009 Dengue Case Classification

**Figure 3:**
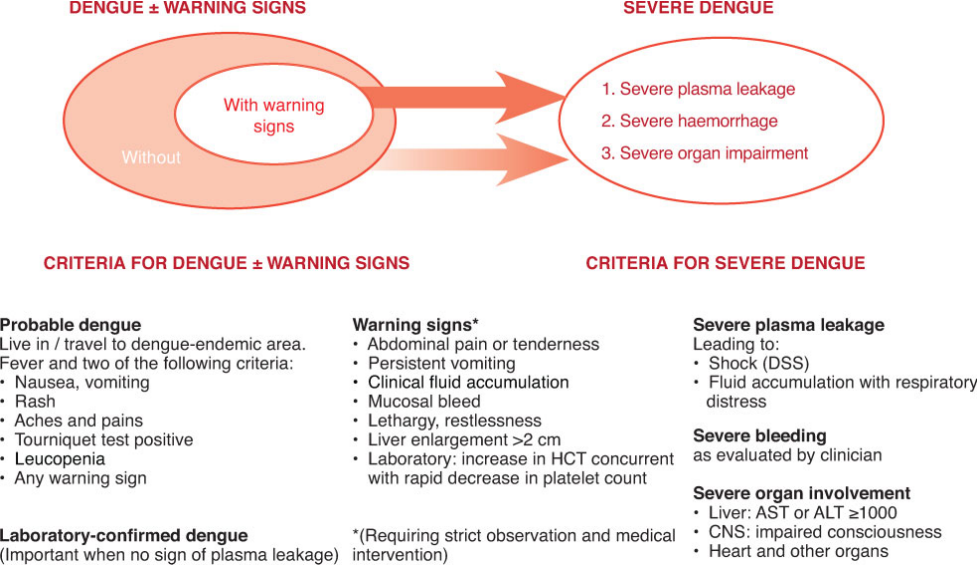
The 2009 revised dengue case classification^6^

The 2009 WHO criteria (Fig. 3) classify dengue according to levels of severity: dengue without warning signs; dengue with warning signs (abdominal pain, persistent vomiting, fluid accumulation, mucosal bleeding, lethargy, liver enlargement, increasing haematocrit with decreasing platelets); and severe dengue (dengue with severe plasma leakage, severe bleeding, or organ failure).^6^ Patients who recover following defervescence are considered to have non-severe dengue, but those who deteriorate tend to manifest warning signs.^6^ These individuals are likely to recover with intravenous rehydration. However, further deterioration is classified as severe dengue, though recovery is possible if appropriate and timely treatment is given.^6^

## Applicability and Perceptions of the 2009 Classification System

The 2009 classification into severity levels is considered to be more sensitive in capturing severe disease than the 1997 guidelines, with observed sensitivities of up to 92% and 39%, respectively.^18,19^ A multi-centre study across 18 countries demonstrated that approximately 14% of cases could not be classified using the DF/DHF/DSS classification, even when strict DHF criteria were not applied, compared with only 1·6% with the revised system (Table 1).^10^ The study also examined acceptance by and user-friendliness to healthcare professionals.^10^ The new classification was particularly useful with respect to triage and management of dengue, reporting during surveillance and for endpoint measurements in dengue clinical trials.6,^10^

**Table 1:** Comparison of the 1997 and revised classifications^10^

DF/DHF/DSS classification by expert reviewer	Revised classification by expert reviewer				Total
	Not classifiable*	Dengue WS negative	WS positive	Severe dengue	
Not classifiable	23 (8·6%)	57 (21·3%)	159 (59·3%)	29 (10·8%)	268 (100%) (13·7% of all)
DF	7 (0·5%)	551 (41·8%)	684 (51·9%)	75 (5·7%)	1317 (100%) (67·1% of all)
DHF (grades 1 & 2)	2 (0·7%)	8 (2·8%)	240 (83·0%)	39 (13·5%)	289 (100%) (14·7% of all)
DSS (DHF grades 3 & 4)	0	0	12 (13·6%)	76 (86·4%)	88 (100%) (4·5% of all)
Total	32 (1·6%)	616 (31·4%)	1095 (55·8%)	219 (11·2%)	1962 (100%)

* Not classifiable, classification was not possible; WS, warning signs

However, problems with the use of the revised classification have also been noted. Additional training for healthcare workers and dissemination of information may be required to remedy any confusion over the changes to the system.^10^

## Other Considerations for the 1997 and 2009 Classification Systems

### Geographical considerations

Local guidance for the diagnosis and classification of dengue, based on the WHO DF/DHF/DSS criteria, varies between countries and regions.^20^ In addition, the incidence by patient age varies according to region.^4^ The resulting inconsistent application of the guidelines highlights the importance of standardising case classification and management. Furthermore, variations in the warning signs defined by the revised classification can be observed in different regions, thus requiring local adaptation of the guidelines in the future.^10^ In fact, although considered particularly valuable for dengue case management and triage, there is concern that the use of warning signs in highly dengue-endemic countries such as Indonesia, can contribute to unnecessary hospital admissions.

### Classification of dengue during an outbreak

Between 1966 and 1997, the application of the original WHO case definition guidelines, dengue morbidity and mortality decreased, with mortality in Indonesia reduced from 45% in 1969 to 1·2% in 2009.^2^ However, during dengue outbreaks in Jakarta, mortality from severe, hospitalised cases increased from 25% of cases with shock in 1988 to approximately 47% of those with severe complications during the 2004 outbreak.^21^ This supports the need to include complications of dengue such as haemorrhage, fluid overload, organ involvement and encephalopathy in the case definition of severe dengue.

### Potential modifications to the 2009 classification system

Some of the concepts, including triage, warning signs and dengue severity, are discussed in the 1997 guidelines^9^ and components of the previous classification may be suitable for clinical practice. Furthermore, research on the revised 2009 classification system is necessary in order to optimise dengue case definitions. There is a need for more precise definition of warning signs to enable optimal triaging for more accurate identification of patients who require hospitalisation as opposed to those who can be treated as outpatients.^10^ Quantitative research into the predictive value of these warning signs on patient outcomes across all affected geographical areas and age groups is also crucial.^10^ There is an additional need to explore the potential barriers to and cost-effectiveness implications of implementing the new classification system.

## Conclusions

The 1997 WHO case classification system for dengue was revised because of differences across the broad geographical areas and the age groups affected by dengue. However, the current 2009 WHO classification has yet to be definitively proved to be effective. The question remains, therefore, whether this latest classification requires further modification. A solution may be to incorporate elements from the 2009 classification of severe dengue into the 1997 guidelines, much of which remains relevant for use. This may be resolved by conducting multi-centre, prospective studies using standardised protocols in Asia and Latin America in a full range of patient age groups.

**Table:** Box 1 WHO 1997 case definitions for DF, DHF and DSS^10^

DF	**Probable**
	• An acute febrile illness with two or more of the following manifestations: headache, retro-orbital pain, myalgia, arthralgia, rash, haemorrhagic manifestations and leucopenia
	*and*
	• Supportive serology (a reciprocal haemagglutination-inhibition antibody titre ⩾1280, a comparable IgG enzyme-linked immunosorbent assay (ELISA, see chapter 49,^10^) titre or a positive IgM antibody test on a late acute or convalescent-phase serum specimen)
	*or*
	• Occurrence at the same location and time as other DF cases
	**Confirmed**
	• A case confirmed by one of the following laboratory criteria:
	– Isolation of the dengue virus from serum/autopsy samples
	– An at least four-fold change in reciprocal IgG/IgM titres to one or more dengue virus antigens in paired samples
	– Demonstration of dengue virus antigen in autopsy tissue, serum or cerebrospinal fluid samples by immunohistochemistry, immunofluorescence or ELISA
	– Detection of dengue virus genomic sequences in autopsy tissue serum or cerebrospinal fluid samples by polymerase chain reaction (PCR)
	**Reportable**
	• Any probable or confirmed case should be reported
DHF	For a diagnosis of DHF, a case must meet all four of the following criteria:
	• Fever or history of fever lasting 2–7 days, occasionally biphasic
	• A haemorrhagic tendency shown by at least one of the following: a positive tourniquet test; petechiae, ecchymoses or purpura; bleeding from the mucosa, gastro-intestinal tract, injection sites or other locations; or haematemesis or melaena
	• Thrombocytopenia [⩽100,000 cells/mm^3^ (100×10^9^/L)]
	• Evidence of plasma leakage owing to increased vascular permeability shown by: an increase in haematocrit ⩾20% above average for age, sex and population; a decrease in the haematocrit after intervention ⩾20% of baseline; signs of plasma leakage such as pleural effusion, ascites or hypoproteinaemia
DSS	For a case of DSS, all four criteria for DHF must be met, in addition to evidence of circulatory failure manifested by:
	• Rapid and weak pulse
	*and*
	• Narrow pulse pressure (<20 mmHg or 2·7 kPa)
	*or manifested by*
	• Hypotension for age
	*and*
	• Cold, clammy skin and restlessness

## References

[1] World Health Organization (2010). Working to Overcome the Global Impact of Neglected Tropical Diseases. First WHO report on Neglected Tropical Diseases.

[2] Bandyopadhyay S, Lum LC, Kroeger A (2006). Classifying dengue: a review of the difficulties in using the WHO case classification for dengue haemorrhagic fever.. Trop Med Int Health..

[3] World Health Organization Dengue and Dengue Haemorrhagic Fever. Fact sheet 117, 2009 [cited 28 November 2011]. www.who.int/mediacentre/factsheets/fs117/en/.

[4] Whitehorn J, Simmons CP (2011). The pathogenesis of dengue.. Vaccine..

[5] Chuansumrit A, Chaiyaratana W, Tangnararatchakit K, Yoksan S, Flamand M, Sakuntabhai A (2011). Dengue nonstructural protein 1 antigen in the urine as a rapid and convenient diagnostic test during the febrile stage in patients with dengue infection.. Diagn Microbiol Infect Dis..

[6] World Health Organization (2009). Dengue: Guidelines for Diagnosis, Treatment, Prevention and Control.

[7] Gubler DJ (1998). Dengue and dengue hemorrhagic fever.. Clin Microbiol Rev..

[8] Guzman MG, Kouri G (2002). Dengue: an update.. Lancet Infect Dis..

[9] World Health Organization (1997). Dengue Haemorrhagic Fever: Diagnosis, Treatment, Prevention and Control, 2nd edn.

[10] Barniol J, Gaczkowski R, Barbato EV, da Cunha RV, Salgado D, Martínez E (2011). Usefulness and applicability of the revised dengue case classification by disease: multi-centre study in 18 countries.. BMC Infect Dis..

[11] Deen JL, Harris E, Wills B, Balmaseda A, Hammond SN, Rocha C (2006). The WHO dengue classification and case definitions: time for a reassessment.. Lancet..

[12] Rahman M, Rahman K, Siddque AK, Shoma S, Kamal AH, Ali KS (2002). First outbreak of dengue hemorrhagic fever, Bangladesh.. Emerg Infect Dis..

[13] Rigau-Perez JG (2006). Severe dengue: the need for new case definitions.. Lancet Infect Dis..

[14] Phuong CX, Nhan NT, Kneen R, Thuy PT, van Thien C, Nga NT (2004). Clinical diagnosis and assessment of severity of confirmed dengue infections in Vietnamese children: is the World Health Organization classification system helpful?. Am J Trop Med Hyg..

[15] Balmaseda A, Hammond S, Perez M, Cuadra R, Solano S, Rocha J (2005). Short report: assessment of the World Health Organization scheme for classification of dengue severity in Nicaragua.. Am J Trop Med Hyg..

[16] Harris E, Videa E, Perez L, Sandoval E, Tellez Y, Perez ML (2000). Clinical, epidemiologic, and virologic features of dengue in the 1998 epidemic in Nicaragua.. Am J Trop Med Hyg..

[17] Alexander N, Balmaseda A, Coelho IC, Dimaano E, Hien TT, Hung NT Multicentre prospective study on dengue classification in four South-east Asian and three Latin American countries. Trop Med Int Health.

[18] Basuki PS, Budiyanto, Puspitasari D, Husada D, Darmowandowo W, Ismoedijanto (2010). Application of revised dengue classification criteria as a severity marker of dengue viral infection in Indonesia.. Southeast Asian J Trop Med Public Health..

[19] Narvaez F, Gutierrez G, Perez MA, Elizondo D, Nunez A, Balmaseda A (2011). Evaluation of the traditional and revised WHO classifications of dengue disease severity.. PLoS Negl Trop Dis..

[20] Santamaria R, Martinez E, Kratochwill S, Soria C, Tan L, Nunez A (2009). Comparison and critical appraisal of dengue clinical guidelines and their use in Asia and Latin America.. Int Health..

[21] Citraresmi E, Hadinegoro SR, Akib AAP (2007). Diagnosis and management of dengue haemorrhagic fever during an outbreak in six hospitals in Jakarta.. Sari Ped Siartri I..

